# “*You feel hopeless when you can't access healthcare*”: International students' experiences of mental health help-seeking through primary healthcare services in Scotland

**DOI:** 10.1080/17482631.2025.2572518

**Published:** 2025-11-02

**Authors:** Ula Kolinska, Rowena Piers, Dimitar Karadzhov

**Affiliations:** a School of Health in Social Science, University of Edinburgh, Edinburgh, UK; b School of Health & Wellbeing, University of Glasgow, Glasgow, UK

**Keywords:** International students, higher education, mental health, transnational help-seeking, primary healthcare, liminality

## Abstract

**Purpose:**

International university students (ISs) experience elevated rates of psychological distress due to the unique challenges of living and studying in a new country. Nonetheless, their utilisation of mental health services tends to be low. This study aimed to explore ISs' experiences of help-seeking via the Scottish primary healthcare services.

**Methods:**

A qualitative design using semi-structured interviews and interpretative phenomenological analysis (IPA) was employed. The sample included nine female ISs from a range of cultural backgrounds, who sought mental health support from the Scottish primary healthcare services within the previous year.

**Results:**

Participants' help-seeking experiences, contextualised within the challenging, liminal reality of living and studying in Scotland as an IS, were hindered by challenges with navigating two healthcare systems simultaneously and culturally-mediated attitudes towards mental health. Positive and negative experiences of patient-GP interactions had a considerable impact on participants' subsequent help-seeking endeavours.

**Conclusions:**

Transnational and relational lenses are key for understanding ISs' help-seeking. Beyond individual factors, help-seeking trajectories hinge on perceived quality of patient-doctor relationships and the accessibility of both local and home-country systems. These findings highlight the need for universities to implement targeted mental wellbeing interventions for ISs, and primary care to improve the quality patient–GP interactions.

## Introduction

### Mental health of international students in the UK

Although concerns regarding student mental health in the UK date back to the 1940s (Crook, 2020), evidence shows that the situation continues to deteriorate despite ongoing efforts to address it (Akram et al., 2023; Lewis & Bolton, 2023; Sanders, 2023). The 2023 Student Academic Experiences Survey (SAES) showed that in a sample of 10,163 UK undergraduate students, 16% reported mental health difficulties (Sanders, 2023). Data collected via validated psychiatric scales is a cause for even greater concern, with reports of 47% university students meeting the criteria of moderate to severe depression, 44% of moderate to severe anxiety and, most alarmingly, 37% being at risk of suicidal thoughts of behaviours (Akram et al., 2023). Overall, the prevalence of mental health symptoms among UK university students is greater than in non-students of the same age (Lewis et al., 2021). Beyond concern for students' wellbeing, this trend is likely to affect students' educational outcomes, since students who do experience psychological distress may face difficulties achieving their full academic potential (Macaskill, 2013), with poor mental health being credited for a significant share of university dropout rates (Bradley, 2017; Brennan, 2022; Neves et al., 2024; Sanders, 2023). Therefore, mental health of UK university students continues to be a major public health challenge which requires urgent attention and action.

These adverse mental health outcomes have been attributed to the multiple challenges which students face in university settings, including social, academic and financial difficulties (Clough et al., 2019; Cooke et al., 2006; Macaskill, 2013; McCloud & Bann, 2019). For international students (ISs), these challenges seem to be amplified (Ansari Lari et al., 2025; Prieto-Welch, 2016); whilst struggling with the typical social, academic and financial challenges of being a student, ISs also face unique stressors associated with living and studying in a new country, such as the UK (Hyams-Ssekasi et al., 2014; McMahon, 2018). These unique stressors—such as cultural differences, communication and language barriers, sense of isolation and loneliness—are often attributed to the process of acculturation, which involves the transition to new socio-cultural and academic environments (Aladegbaiye et al., 2022). Rooted in the works of Berry (1997), acculturation research frames migration experiences in terms of psychological and socio-cultural changes that occur during contact between two distinct cultures. These changes are accompanied by acculturative stress and involve a range of strategies to resolve the tension between wanting to maintain one’s heritage, culture and identity, whilst re-establishing their lives in the host country's cultural context (Berry et al., 2016; Kristiana et al., 2022). Acculturative stress in particular has been linked to negative mental health outcomes among ISs, with research demonstrating a positive correlation between severity of acculturative stress and psychological distress (Feba & Sumathi, 2018; Gebregergis, 2018; Liu et al., 2016). Furthermore, acculturative stress appears to mediate the relationship between acculturation and psychological distress (Castillo et al., 2015).

Nevertheless, some scholars criticise the acculturation lens as insufficient to capture the lived, subjective and processual dimensions of migration (e.g., Ngo, 2019; Rudmin, 2003; Sapountzis et al., 2024; Schwartz et al., 2010). Meanwhile, the anthropological notion of liminality offers an alternative approach to conceptualising ISs' experiences, that addresses the deficiencies of the acculturation approach. Liminality describes an ambiguous, suspended status, an uncertain state of being *“betwixt-and-between*” (Turner, 1979, p. 465) that one enters when transitioning from one life stage to another. Unlike acculturation's rigid categories, liminality embraces the complexity, instability, and active negotiation of identity, agency and belonging as ongoing processes. This approach seems better suited to foreground the profoundly transitional character of ISs’ migration, rather than framing it a simply a matter of assimilating or integrating into a static host society. It thereby shifts the analytic lens away from essentialising difference to understanding the common, yet deeply individualized, processes of navigating change that ISs undergo when transitioning to living and studying abroad (De Luca Picione et al., 2025; Ngo, 2019; Simich et al., 2009). Therefore, this paper will draw on liminality to conceptualise ISs' lived experiences of transitioning to living and studying in Scotland, allowing a more nuanced understanding of the associated mental health difficulties and help-seeking that extends beyond existing acculturation models.

### Help-seeking for mental health difficulties among ISs

Help-seeking is “*an adaptive coping process that is the attempt to obtain external assistance to deal with a mental health concern*” (Rickwood & Thomas, 2012, p. 180). Within the Scottish National Healthcare System (NHS), general practitioners (GPs) are the first formal point of contact regarding mental health concerns (i.e., primary healthcare), making diagnoses, offering treatments and referring to mental health specialists (Mind, 2017). Although students are a high-risk group for developing mental health difficulties, their utilisation of mental health services is low (Osborn et al., 2022). Broglia et al. (2021) found that out of 1,516 UK students, both domestic and international, almost a half avoided seeking help for their mental health concerns. Clough et al. (2019) point out that this reluctance to seek help is even more pronounced among ISs, with the IS status being a significant predictor of poorer help-seeking attitudes in the studied sample. For those who eventually decide to seek help, primary healthcare is unlikely to be their first choice (Frampton et al., 2022). To better understand the low rates of mental health help-seeking among ISs, contextualised within the unique challenges associated with living and studying in a foreign country, ISs' experiences of help-seeking via primary healthcare services need further exploration.

There are several theoretical approaches to conceptualise the help-seeking process, including help-seeking barriers and facilitators. For instance, the behavioural model of health service use (Andersen, 1995) focuses on the predisposing, enabling and need factors that influence healthcare availability and access. Though useful to determine help-seeking patterns on a population level, this model does not lend itself to explore the experiential, idiosyncratic dimensions of help-seeking (Babitsch et al., 2012; Lederle et al., 2021). Alternatively, the health behaviour belief model (Rosenstock, 1974) describes help-seeking endeavours as determined by one's beliefs regarding their susceptibility to a condition, whether the condition would have serious consequences, benefits of seeking help outweighing the barriers, along with exposure to cues to action and feeling confident in their ability to perform the action. However, this model’s focus on cognitive factors diminishes the role of emotional, social, and cultural influences on help-seeking behaviours (Davidhizar, 1983; Thomas, 1995; Yarbrough & Braden, 2001). A third approach—Rickwood et al. (2005) help-seeking framework—was developed specifically to describe mental health help-seeking pathways, and maps the sequential psychological and social stages of help-seeking. These include one's awareness of their mental health problems, ability to express their symptoms and need for support, recognition of available sources of help and finally willingness to reach out to these sources. Rickwood et al. (2005) conceptualisation appears to be best suited to explore the experiential dimensions of help-seeking, providing insight into why individuals may hesitate or progress through stages of help-seeking. For this reason, it was chosen as most relevant to frame the present exploration of ISs' experiences of help-seeking through primary healthcare services while living and studying in Scotland.

The underutilisation of mental health services by ISs suggests that there are barriers hindering help-seeking in this population. One of such barriers may be ISs' unfamiliarity with the NHS (i.e., perceived unavailability of sources of help; Rickwood et al., 2005), with past research showing that they find it challenging navigating the NHS to access mental health support (Frampton et al., 2022; Maguire & Cameron, 2021). Another well-recognised barrier to help-seeking among ISs is mental health stigma—the negative attitudes, beliefs, and stereotypes regarding mental health issues (AthinarayananRao et al., 2025; Dombou et al., 2023; Hei et al., 2024; Subu et al., 2021), embedded within culture (Gopalkrishnan, 2018; Koschorke et al., 2017). For instance, in some Arab cultures, mental illness may be attributed to culturally related beliefs such as magic, witchcraft or divine punishment, and so succumbing to it could be perceived as a moral failing (Ahad et al., 2023; Fekih-Romdhane et al., 2023). Meanwhile, Western Balkan culture strongly values self-reliance and privacy, meaning that struggling with mental health is seen as a personal weakness and a source of shame (Hyseni Duraku et al., 2024). Even if not personally subscribing to such cultural beliefs, people experiencing psychological distress may be aware of the negative mental health attitudes prevailing on a cultural level (i.e., perceived stigma; Bracke et al., 2019; Tesfaw et al., 2020), which they may then internalise and express as their own attitudes (i.e., personal stigma; Latalova et al., 2014). Perceived and personal stigma are associated with reluctance to seek help for mental health difficulties among both domestic and international students, thereby acting as a help-seeking barrier (Cage et al., 2020; Jennings et al., 2015; Pedersen & Paves, 2014). It may hamper help-seeking at all stages, reducing recognition of need, negatively affecting ability to communicate their symptoms, distorting perceptions of service availability and directly inhibiting readiness to seek or accept help (Dombou et al., 2023; Frampton et al., 2022; Rickwood et al., 2005; Takeuchi & Sakagami, 2018). Crucially, research shows that some IS populations exhibit greater mental health stigma compared to domestic students, resulting in greater help-seeking difficulties (e.g., LaMontagne et al., 2023; Maeshima & Parent, 2022).

Nevertheless, by attributing low help-seeking rates among ISs primarily to their unfamiliarity with the NHS and stigmatising attitudes, studies overlook remaining factors affecting help-seeking, for instance systemic and relational factors. Such research fails to acknowledge that help-seeking is not a one-sided process but an interaction between doctor and patient (Cocoros et al., 2019). As the first point of access to mental health support in Scotland is the GP (Mind, 2017), the relational dynamics that exist within a patient-GP interaction may be crucial in determining the patients' subsequent help-seeking endeavours. First, the quality of the patient-GP interaction itself has been linked to help-seeking outcomes (Buszewicz et al., 2006). A perceived positive patient-GP interaction in mental health consultations was found to predict positive treatment outcomes (Priebe et al., 2017; Wampold et al., 2023) and facilitate further help-seeking (Buszewicz et al., 2006; Parker et al., 2020a; Tunks et al., 2023). Secondly, studies show that GPs may also express stigmatising attitudes towards patients with mental disorders (i.e., professional stigma; Subu et al., 2021; Vistorte et al., 2018). Professional stigma exhibited by GPs within the medical interaction can diminish the quality of the patient-GP interaction, and so hamper patients' help-seeking endeavours (Blixen et al., 2016; Knaak et al., 2017; Nyblade et al., 2019; van Boekel et al., 2013). Despite this, to the research team's best knowledge, no research into the role of patient-GP interaction in shaping help-seeking endeavours has been conducted among ISs. Therefore, the present study strived for a more comprehensive perspective on the factors affecting help-seeking by exploring how ISs conceptualised the patient-GP interactions throughout their experiences of seeking help for their mental health concerns through the Scottish primary healthcare services.

### Present study

The current study aimed to examine ISs' experiences of seeking help for their mental health concerns via the Scottish primary healthcare services, whilst navigating the transition to living and studying in Scotland. Based on the previously discussed literature, ISs' experiences and perceptions of the patient-GP interactions were of particular interest.

## Methods

### Design

Given the exploratory character of the research aims, this study employed an interpretative phenomenological analysis (IPA) design. This qualitative approach considers a phenomenon as a lived *process* situated within and shaped by the unique world each participant inhabits (i.e., lifeworld; Gorichanaz et al., 2018; Smith et al., 2022). This theoretical orientation makes IPA particularly suitable to investigate help-seeking, which is inherently a multi-dimensional, idiosyncratic process (Rickwood & Thomas, 2012)*.* This is especially the case for help-seeking experiences, which are situated within the unique lifeworld of living and studying in Scotland, giving them a “*complex, ambiguous and emotionally laden*” character (Smith & Osborn, 2015, p. 41).

### Participants and recruitment

Purposive sampling was employed to identify a group of participants that possessed qualities relevant to the study objectives (Etikan et al., 2015). This sampling approach is advised by Smith et al. (2022), to ensure that participants represent the specific perspective of interest, rather than a wider population. Then, convenience sampling was used to recruit them from a readily available pool (Garvey et al., 2021). Participants were recruited through online adverts and poster boards around campus. Individuals who expressed interest in participating were screened against the eligibility criteria (Table I) and, once their eligibility was confirmed, were handed a copy of the participant information sheet and given the opportunity to ask questions. Having the questions answered to their satisfaction, they signed a consent form and completed a demographic survey.

**Table I.** Eligibility criteria.

• Between 18 and 24 years old • International (non-UK) university student • The primary reason they moved to the UK was to attend university • English as not their first language • Visited a GP in the UK regarding their mental health concerns within the past year

The sample consisted of nine female participants from a diverse range of backgrounds (mean age of 22.67 years; *SD* = 1.22; Table II). Four were undergraduate students, four postgraduate students and one PhD student, all with the experience of being an IS as defined in the eligibility criteria (Table I). Participants' mean total length of time living in the UK was 2.89 years (*SD* = 2.31) and ranged between seven months and six years. All had previously reached out to their GP in the UK regarding their mental health concerns within 12 months prior to the interview.

**Table II.** Demographic characteristics of participants.

Pseudonym	Gender	Age	Year of study	Level of study	Country of origin	Years in the UK
Monika	Female	21	3	Undergraduate	Poland	6.00
Sylvia	Female	24	1	Postgraduate	Bulgaria	0.58
Nina	Female	24	1	PhD	Croatia	5.50
Anna	Female	23	1	Postgraduate	Mexico	0.67
Sonia	Female	22	1	Postgraduate	India	0.58
Zahra	Female	21	3	Undergraduate	Jordan	3.00
Kara	Female	22	4	Undergraduate	Italy	4.00
Meera	Female	24	1	Postgraduate	India	0.67
Lucia	Female	23	5	Undergraduate	Italy	5.00

### Data collection

The one-on-one semi-structured interviews were conducted by the first author between April and May 2023. This interviewing approach allows the researcher to obtain rich, in-depth accounts of the participants' experiences, to capture their perspectives of the phenomenon of interest (DeJonckheere & Vaughn, 2019; Smith et al., 2022). The interview schedule (Supplementary Material 1) was developed based on the research aims, as well as past studies exploring people's experiences in primary mental health services (Gask et al., 2003; Leahy et al., 2018). The interview schedule followed a temporal structure, as suggested by Smith et al. (2022), but was flexibly applied to allow participant to lead the narrative. After opening questions aimed at establishing rapport, they were asked about their experiences of reaching out for help and setting up the first appointment. Then, the researcher probed their thoughts and feelings during the appointment. Finally, they were asked how they felt after the appointment and how they rated its effectiveness. The interviews lasted between 32 and 56 minutes. Participants were reimbursed with a £ 20 Amazon voucher. All interviews were audio-recorded and transcribed verbatim.

### Ethics

Ethics approval was granted by the University of Glasgow Ethics Committee (200220209). Informed consent was confirmed in writing prior to participation. Although participants' mental health diagnoses were not enquired about, due to the interviews' participant-led character, participants could disclose this information had they wished to. During the interviewing process, the researcher remained mindful of the participants' wellbeing, paying additional attention to any symptoms of distress (Draucker et al., 2009). In case these were to occur, a distress protocol was created in advance (Whitney & Evered, 2022). No visible signs of distress appeared during interviews, and participants reported no negative effects at the conclusion of the interview. After the interview, participants were provided with a debriefing sheet which included contact details to mental health helplines.

In the transcription process, to protect participants' identities, they were given pseudonyms and any potentially identifying information was omitted. The interview recordings were deleted after transcription and the transcripts were stored securely in a password-protected file.

### Data analysis

Analyses were conducted by the first author using IPA (Smith et al., 2022). First, each transcript was read and re-read whilst making initial comments, to immerse oneself in and empathetically engage with the data (Eatough & Smith, 2008). Then, ensuring that the participants' idiographic experiences were the focus of the analysis and bracketing the researcher's pre-existing knowledge and experiences, comprehensive exploratory notes were made line-by-line. These notes manifested on different levels of interpretation (Smith et al., 2022). Descriptive notes summarised the core elements of participant's help-seeking experiences, as well as how these experiences were interpreted by participants within their lifeworld. Linguistic comments focused on how participant used language to convey their interpretation of the phenomenon of interest. Conceptual notes interrogated the participant's overarching understanding of the matters discussed on a more abstract level. Third, using NVivo (Version 12; Lumivero, 2015), the exploratory notes were summarised into experiential statements which captured the “*experiential core*” (Smith et al., 2022, *p*.87) of their help-seeking journeys. Next, by clustering and compiling experiential statements, Personal Experiential Themes (PETs) were created. These four steps were repeated for each interview. Finally, looking for patterns of convergence and divergence between PETs across cases, Group Experiential Themes (GETs) were developed. In this final stage, the following criteria were applied: relevance in answering research questions, degree of similarity and individual idiosyncrasy, experiential richness and emotional resonance (Alase, 2017; Nizza et al., 2021).

### Rigour and reflexivity

The first author remained attentive of the role that their own preconceptions may have had in the process of data analysis (Dodgson, 2019). An IS with past experiences of seeking help for mental health difficulties through the Scottish primary healthcare services, the researcher was able to adopt an insider's perspective and empathetically engage with participants' experiences (i.e., hermeneutics of empathy; Smith et al., 2022). Meanwhile, keeping reflective notes and analytic memos aided in acknowledging and bracketing these preconceptions at the more interpretative stages of analysis, ensuring that the final results are grounded in the participants' experiences rather than those of the researcher (Chan et al., 2013).

To further enhance rigour of analysis, the first and third authors collaboratively refined the preliminary analysis outcomes during regular supervision meetings. UK presented initial GETs, experiential statements, and supporting quotes to DK, who independently assessed them, noting any ambiguities, tensions or discrepancies. These analytical challenges were then discussed until UK and DK reached consensus and made necessary modifications. This iterative process continued until the final set of GETs was agreed upon. Then, an independent researcher (RP) was consulted and the same procedure of collaboratively refining the analysis was followed. Throughout this process, researchers navigated analytical challenges by ensuring that the findings convey a coherent experiential narrative rooted in ISs' own words, incorporating both convergent and divergent experiential statements to honour IPA's idiographic commitment. This approach allowed to achieve analytical depth of findings (Nizza et al., 2021).

The rigour of reporting was pursued by adhering to the COREQ (Consolidated criteria for Reporting Qualitative research guidelines; Booth et al., 2014; Tong et al., 2007).

## Results

**Figure 1.**
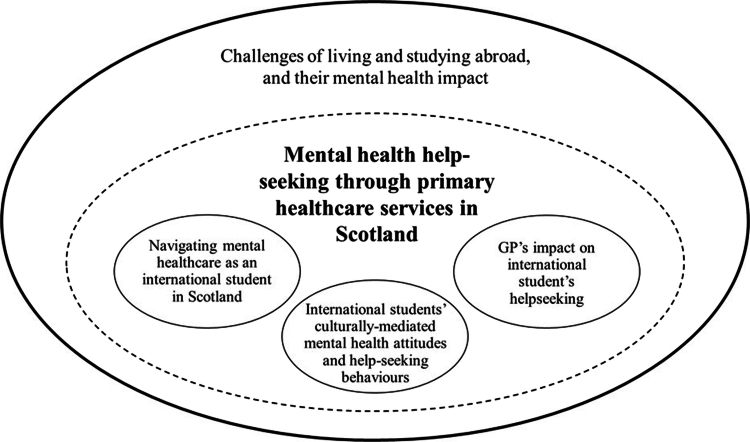
Conceptual map illustrating relationships between GETs.

The GETs which emerged from the analysis and were relevant to the research aims are summarised in Table III and Figure 1.

**Table III.** Summary of the IPA results.

Group experiential themes (GETs)	Experiential statements
Challenges of living and studying abroad, and their mental health impact	• Transitioning to living and studying abroad was a “*big step*” • *“It* *'s not a fairy tale anymore”*
Navigating mental healthcare as an international student in Scotland	• “*Trying to navigate*” a new healthcare system • *“I also have a GP in Italy”*
International students' culturally-mediated mental health attitudes and help-seeking behaviours	• “*Stigma about speaking*” about mental health • “*I think that I just transferred that into here”*
GPs' impact on international students' help-seeking	• “*A shift in culture*” • “*She was very dismissive*” • “*The doctor's behaviour was nice*” • *“I might not reach out to the GP”*

### Challenges of living and studying abroad, and their mental health impact

**Transitioning to living and studying abroad was a “*****big step*****”.** Participants conceptualised their experiences of migrating Scotland as a major transition. For Kara, “*the first couple of months were tough*”. Interestingly, for Anna, this “*big step*” required “*a lot of growing up*”, suggesting that this transition had a dual meaning—adjusting to living and studying in a new country but also transitioning into being an independent adult. The dual nature of this transition was also characterised by Zahra, who pointed out that the universally difficult experience of entering adulthood, was further “*augmented*” by migrating to another country:

“*As well as just regular growing pains in your early twenties. I feel like that's already a very weird time (…). Uhh, but being like away from my family and away from my country and everything, just (…) made things way more confusing.*”—Zahra

***“It's not a fairy tale anymore”.***When reflecting upon their experiences as ISs in Scotland once they “*settled in*”, some participants summarised it in mostly positive terms. As Kara put it:

*“Once I settled in it was fine, it's been fine for a long time”*—Kara

However, for most the day-to-day reality of living and studying in Scotland had not been without its challenges, subsequently affecting their mental health. Unlike Kara, Nina and Sonia were initially excited about the transition to living and studying in a new country, and it was not until they settled in that they started experiencing mounting challenges and their mental health deteriorated. Although Nina had been living in Scotland for over five years, and Sonia for only few months, they seemed to be equally disenchanted about the reality of living and studying in Scotland.

“*When I arrived here, I was like very happy. (…) But when you are trying to live, more and more days pass by, the reality kicks in, it's not a fairy tale anymore.”*—Sonia

The challenges experienced by participants overlapped across many domains, mainly academic, social and financial. Adjusting to the demands of higher education in Scotland made them feel “*pressured*” and “*overwhelmed*”. To cope with the academic challenges, Anna gave up activities that helped her maintain mental wellbeing, e.g., socialising. Sonia and Nina sacrificed their social life due to financial difficulties. The academic and financial obstacles to social life were particularly detrimental, since many participants already felt that, by migrating to Scotland, they lost support networks (a “*safe net*”) they used to rely on in their home countries. Consequently, they felt “*alone*” and “*vulnerable*”, which made coping with the challenges they faced harder, exacerbating the detrimental effect of academic, social and financial pressures on their mental health.

“*Um, for example in Mexico, if I needed to talk with someone, (…) I could always call my friends and meet them. And I knew they would be there for me (…). But here I don't have that.*”—Anna

Notably, although most participants acknowledged the “*very challenging mentally*” reality of living and studying abroad, Anna mentioned that it was also “*worth it*”, because of the positive impact of studying here on her professional prospects.

### Navigating mental healthcare as an IS in Scotland

***“Trying to navigate”*** **a new healthcare system.** Cognisant of how the challenges of adjusting to living and studying in Scotland impacted their mental health, participants decided to seek mental health support. However, they were familiar with neither the mental health resources available in Scotland, nor how to access them.

“*Because if you moved to a different country, you don't know what is the healthcare system*.”—Sonia

As such, in addition to struggling with day-to-day aspects of living and studying in a new country, they had to face the uncertainty of “*trying to navigate*” a new healthcare system. This experience was underscored with a sense of frustration, confusion and hopelessness.

“*It's just that looking for mental health resources can be very frustrating, along with what you're already (…) going through.*”—Zahra

*“You feel hopeless when you can't access healthcare.”*—Sonia

***“I also have a GP in Italy”.*** Participants' previous treatments and medical documentation were based in their home countries, and their time in Scotland limited. Therefore, migrating to Scotland was not only associated with learning to navigate a new healthcare system, but rather balancing two systems simultaneously to ensure a continuity of care.

“*I spend like my summer in Italy. So, it's not like (…) I can just have [a GP] here. I naturally like would have to have two (…)*.”—Lucia

Two participants mentioned that instead of seeking help in the UK, they attempted to continue the help-seeking journey they had started in their home countries. Lucia and Sonia used mental health support back home but had to stop upon migrating to Scotland. For Sonia, this mental health support being “*suddenly*” absent made it harder to cope with the distress she experienced during that difficult transition. Both Lucia and Sonia contacted their GP in Scotland to get medication they were prescribed back home. Both were denied.

Lucia and Sylvia talked about how accessing certain services in Scotland required additional efforts of contacting the doctors back in their home countries, translating the documents and then submitting them in Scotland. For Sylvia, this added effort discouraged her from pursuing certain services altogether:

“*But since all the proof is in Bulgaria, I thought ‘Well, I'm not doing that’.*”—Sylvia

Although Lucia presented her GP with translated test results from Italy, the GP had her re-take these “*invasive*” tests. Lucia found it particularly frustrating, attributing it to Scottish GPs “*distrusting*” patients and doctors from other countries.

### ISs' culturally-mediated mental health attitudes and help-seeking behaviours

***“Stigma about speaking”*** **about mental health.** Participants recognised the prevailing negative attitudes towards mental health, acknowledging the “*shame around asking for help*” and the “*stigma about speaking*” about one's mental health struggles. Notably, most participants saw these stigmatising beliefs as a part of their respective cultures. For example, Sonia talked about how mental illness is seen in India as “*going mad*” or “*psycho*”. Nina described how in the “*Balkan culture*” people with mental illnesses are seen as a “*burden*”, as someone “*crazy*” or “*incompetent*”.

Thus, based on ISs' accounts, their mental health attitudes may have originated from wider cultural attitudes towards mental health and help-seeking. Some made statements which could indicate that ISs have internalised these attitudes and expressed them as personal stigmatising beliefs, with Zahra, for instance, diminishing her mental health struggles as “*outlandish*” and the act of vocalising them as “*trauma dumping*”.

***“I think I just transferred that into here”.*** The stigmatising mental health beliefs were evident in participants' accounts of seeking mental health support through the Scottish primary healthcare services, with the mental health attitudes they expressed translating into actual help-seeking behaviours (or lack thereof).

For instance, after recounting how in Italy *“there's a bit of a shame around asking for help”*, Lucia described how she herself was not sure if mental health is “*important enough*” to seek support, because unlike physical concerns, mental difficulties are “*intangible*” and may be seen by GPs as “*less important*”. During the appointment, she did not know how to “*express*” her mental health concerns to the GP, which is why she started by talking about her physical health, and only then, “*offhandedly towards the end*” disclosed her psychological distress.

Meanwhile, Zahra, who said that in Jordan, “*unless there's something physically wrong with you, then there’s no reason to seek help”*, explicitly acknowledged that this culturally-mediated belief had influenced her own help-seeking endeavours (“*I think I just transferred that into here*”). Not knowing if using the primary health services for mental health alone was “*justified*” and not simply “*misusing the service*”, she did not start seeking mental health support until her mental state began affecting her physical health. She was worried that GPs would not consider her psychological distress to be a “*real thing, if, if they would have taken it seriously, if it wasn't physical*”.

“*That's why I kind of stressed my physic-, the physical aspects that manifested.*”—Zahra

Therefore, participants' mental health beliefs affected their help-seeking endeavours by making them see mental health as a not serious matter. Consequently, they were hesitant to reach out to primary healthcare services and disclose their mental health concerns to GPs.

### GPs' impact on ISs' help-seeking

***“A shift in culture”.*** Some participants noted how normalised and even encouraged mental health help-seeking seems to be in Scotland.

“*Coming here, I, I definitely felt like a shift in culture when it comes to mental health (…). A lot of people talk about it and other people say how important it is*.”—Zahra

However, based on their own experiences, ISs described how this “*shift in culture*” was not reflected in how the primary healthcare providers in Scotland perceived their mental health difficulties. Both Meera and Nina felt that, within the primary healthcare system in Scotland, mental health was not “*perceived as serious*” as opposed to physical health concerns. As such, they believed that the primary healthcare services only considered more severe mental health conditions as serious and requiring treatment.

***“She was very dismissive”.*** When recounting their interactions with GPs, participants reflected on the GPs' demeanour, which mirrored the mental health attitudes within the primary healthcare system.

Participants mentioned how, in going into their GP appointments, they wanted to talk to someone and be listened to, hoping that GPs would acknowledge their mental distress, showing sympathy and compassion. Instead, six participants stated that, after they disclosed their mental health concerns in the appointment, GPs were “*cold*”, “*detached*”, “*impersonal”, “blunt”, “robotic”.* This impersonal approach was compared by Nina and Monika to “*reading (…) off a script*”, following a “*protocol*”. Participants felt like these interactions were “*rushed*”, with GPs “*wanting (…) the appointment over with*”. Rather than compassionately acknowledging the participants' mental distress and their preferred solutions, participants felt that GPs asked only few questions regarding their condition, kept “*directing*” the conversation and even “*cutting off”* the patients.

Meera and Lucia, who had had prior experiences of discussing physical health issues with GPs, felt that their physical concerns were taken “*much more seriously*” than mental health issues. Their physical health issues were “*well looked after*”, whereas “*the mental problems were kind of cast aside*”. In contrast to the “*cold*”, “*impersonal*”, “*rushed*” approach towards participants' mental health concerns, participants described how the GPs acknowledged their physical health problems, asked many questions, provided reassurance. This perceived difference in the GPs' attitude towards mental versus physical health concerns reflects what the participants noticed about the Scottish primary healthcare services in general, seeing mental health as less “*serious*”.

Moreover, Anna, Nina and Meera said that the only questions they were asked, were to assess the severity of their mental health state and verify whether they are indeed in need of help. For Nina, the feeling of having to prove that her struggles are indeed valid was a particularly degrading process:

“*Like you have to hit a certain number of, uh, checkboxes to get through to the next stage. Um, it's almost like a, a video game, you know? [mockingly] Am I depressed enough to get through to the, to the next round of seeing my GP?”*—Nina

Anna felt very vulnerable in that moment, saying that:

“*I was really putting myself in there about my troubles, and the person didn't seem very empathetic about it. Like, felt like it was just, just another patient*.”—Anna

Instead of being treated like “*just another patient*”, Lucia wanted her GP to see her as a “*person with a problem*”. Anna called this the “*human approach*”, which to her meant GPs “*showing empathy*”, acknowledging “*that you're a human and [the GP] understands your feelings*”.

What solidified the sense of dismissal, were the unsatisfying solutions that GPs offered to them. Anna felt that her mental health concerns were entirely invalidated, with the GP attributing them to the weather and saying that no treatment is necessary. Lucia and Meera were offered contact details to external mental healthcare providers.

“*I felt like she just gave me something to, which was a flyer, to just kind of say ‘Oh here is this thing and now we, we can finish the appointment’*.”—Lucia

Nina and Monika went to their GPs hoping for a counselling referral. Instead, the GP said that they don't consider counselling as “*helpful*” and prescribed them medication. Nina found this particularly disappointing, saying that:

“*I think you expect that (…) the health service is going to do something to try and take care of you and it just felt very like ‘Well, this is the easiest and cheapest solution for us, so we're going to send you home with a box of pills and hopefully that works’, you know?*”—Nina

Thus, participants perceived the solutions offered to them by GPs not to be driven by the patient's good, but instead by convenience for the primary healthcare services.

***“The doctor's behaviour was nice”.*** In contrast, Sonia and Zahra did experience what Anna defined as “*human approach*”. In Zahra's experience, her GP was “*caring*” and “*sympathetic*” after she disclosed her mental health concerns. She found this reaction particularly “*encouraging*” and “*reassuring*”, which was particularly important to her considering her initial reluctance to disclose her mental health difficulties and worry that they would not be seen as serious enough to grant GPs' attention.

Similarly, Sonia was particularly appreciative of the way her GP “*seemed concerned with what she is going through*”, “*trying to understand*”, “*knowing the gravity of the situation*”, and that the GP took her mental health concerns as serious and valid. Consequently, she felt not only listened to and understood, but most importantly that she would be cared for and not dismissed. In that sense, the doctor's demeanour bolstered her confidence in the primary healthcare services and the feeling that she can rely on them for mental health support.

***“I might not reach out to the GP”.*** Participants' perceptions of their GPs' demeanour during patient-GP interactions, and the mental health attitudes this demeanour conveyed, had a long-term impact on their further help-seeking endeavours.

For most participants, their experiences of dismissal and invalidation often led to a disenchantment with the Scottish primary healthcare services. Feeling like they cannot “*expect anything”* from the system or “*trust*” the GP to provide the help they need and deserve, they no longer saw it as a viable help-seeking pathway. As Nina and Kara put it:

“*Um, so yeah, in that sense, kind of bittersweet, just knowing that I shouldn't be expecting anything from the NHS and I’ve kind of made my peace with that.*”—Nina

“*If, if I do have any further distressing events happen in my life, I might not reach out to the GP. And I know it's sad but it's just. I, I don't want to go through that again*.”—Kara

Only Sonia, who described her experiences with the GP in mostly positive light, later felt that she can rely on the service for support. She recounted how a “*heavy weight that was inside her heart*” was “*lifted away*”, once she realised that the GP is “*ready to help*” her and she is no longer “*alone*” in trying to cope with her mental health.

Feeling like they could not rely on the Scottish primary healthcare services for mental health support, some participants took matters into their own hands. For Anna and Kara, this meant coping with their mental health issues on their own. After being told by the GP that her mental health concerns are not severe enough to justify treatment, Anna “*started to do things more proactively to feel better, like take walks, cook*”. When Kara was offered antidepressants instead of her preferred treatment—therapy—she decided she would rather “*deal with it on her own*”. Meanwhile, Nina and Lucia chose alternative help-seeking pathways to receive the support they were hoping for. Whereas Nina opted for private counselling, Lucia turned to a psychiatrist in Italy.

## Discussion

This IPA study explored the nuanced nature of nine female ISs' help-seeking endeavours via the Scottish primary healthcare services, contextualised within the uniquely challenging reality of living and studying abroad. It revealed complexities related to navigating healthcare settings by ISs—the confusion of navigating a novel healthcare system, whilst simultaneously continuing to use services in their home countries. These experiences of navigating healthcare settings in search of mental health support were hindered by ISs' attitudes towards mental health. Crucially, the findings point towards the role of patient-GP interaction shaping how ISs seek help for mental health difficulties, thus advancing our understanding of help-seeking barriers in this population. Nonetheless, mindful that generalisability is not the aim of IPA, presented findings speak to the study participants' experiences and should be understood within the described context.

### Mental health of ISs in Scotland: acculturation and liminality

Consistent with past research (Clough et al., 2019; Cooke et al., 2006; Macaskill, 2013; McCloud & Bann, 2019), ISs in this study experienced living and studying in Scotland as a transition which was marked by multiple challenges, including academic, social and financial. While not unique to ISs, what emerged as unique compared to home students is how these challenges are “*augmented*”, as one participant put it, by cultural differences and the sense of loneliness they experience upon migrating to another country. These findings are corroborative of past studies of IS populations in the UK (Hyams-Ssekasi et al., 2014; McMahon, 2018), while extending this to a Scottish context (Cogan et al., 2024). For instance, Cogan et al. (2024) found that Asian ISs experienced alienation and loneliness, struggling to reconcile the cultural differences between their home country and Scotland. The researchers conceptualised ISs' transition to living and studying in Scotland and the associated challenges as acculturation, and the negative impact of these challenges on mental health as acculturative stress (Cogan et al., 2024; Kristiana et al., 2022). The present study revealed that participants experienced stressors that may be construed as acculturative stress. This supports a large body of quantitative research that identified a positive relationship between acculturation and psychological distress, mediated by acculturative stress (Castillo et al., 2015; Feba & Sumathi, 2018; Gebregergis, 2018; Liu et al., 2016). However, while acculturation is applicable to explain the mental health difficulties that ISs faced when living and studying in Scotland, their overall experience can be better understood through the liminality approach.

The experiential lens of this investigation emphasised the deeply transitional and even transformative character of ISs' migration to Scotland, which better aligns with the concept of liminality rather than the rigid categories of acculturation. Whilst navigating the transition to living and studying in Scotland, ISs found themselves “*overwhelmed*” and “*vulnerable*”, in a state of struggling to navigate the precarious reality of living away from their home countries whilst recognising the opportunities that migrating to Scotland allowed them. As such, their migration was a liminal experience, an occasion of “*significant transition, passage or disruption*” (Stenner & Stenner, 2017, p. 14). In line with van Gennep et al. (1960) and Turner (1969), ISs left behind the pre-liminal state of living in their home countries, finding themselves in a spatiotemporal threshold state which they described as “*tough*” and “*confusing*”, until some of them eventually “*settled in*”, emerging into the post-liminal transformed, having gained education which broadened their professional prospects.

Furthermore, ISs noted that their transition to living and studying in a foreign country required “*a lot of growing up*”. This is consistent with previous literature, in which entering higher education is described as a transitionary period, marking the end of adolescence start of adulthood (Ansari Lari et al., 2025; Gulliver et al., 2023; Young et al., 2020). It appears then that ISs found themselves in a dual liminal space (Hajati, 2010; Kirk et al., 2017)—transitioning not only to new cultural experiences, while also navigating the turbulent period before achieving adulthood. According to Marshall et al. (2019) dual liminality model of cancer and adolescence, the two liminal states do not merely co-exist but exacerbate one another, producing a unique set of challenges and inhibiting the young person's progress through either. While experiencing very different challenges, ISs similarly described their experiences of transitioning to living and studying in Scotland as made more difficult by the “*growing pains in your early twenties*”. This also aligns with Coleman, 1974; Coleman, 1978 focal theory, proposing that, during the complex psychosocial transition into adulthood, young adults tend to cope by focusing on individual challenges in turn. When confronted with multiple challenges at once, as was the case for ISs in the present study, they may struggle to pace their transitions, which can lead to greater distress (Hollingworth & Jackson, 2016). Ultimately, these findings further underscore the complex nature of ISs' experiences of transitioning to living and studying in Scotland, the resulting mental distress and help-seeking. This suggests the applicability of dual liminality to unravel these complexities and even extend our current understanding beyond existing acculturation models.

### Barriers to ISs' help-seeking in the Scottish primary healthcare services

This study revealed the difficulties ISs experienced in understanding and navigating the NHS—a common help-seeking barrier among some migrant populations (Akhtar et al., 2022; Straiton & Myhre, 2017). The participants reported feeling unfamiliar with the NHS and the mental health resources available within it. This unfamiliarity with the help available through the NHS was “*frustrating*” and “*overwhelming*”, and, in line with Rickwood et al. (2005), it disrupted their help-seeking endeavours by negatively affecting their perceptions of availability of sources of help. Likewise, Maguire and Cameron (2021) and Frampton et al. (2022) found that navigating the NHS was confusing, particularly for ISs, thus hindering mental health help-seeking.

What was novel is how ISs in the present study experienced an additional challenge of navigating two healthcare systems simultaneously. Although not explored specifically among ISs, transnational healthcare use and cross-border help-seeking have been documented among migrants more broadly (Kemppainen et al., 2018; Nielsen et al., 2012; Troccoli et al., 2022). In the present study, some ISs had received mental health treatments in their home countries and struggled to continue them after migrating to Scotland. Similar experiences were described in case studies by Troccoli et al. (2022), where Polish migrants in the UK either struggled to continue treatments they were prescribed before migrating to the UK and occasionally visited Poland to receive services which they could not access in the UK. However, a facet of transnational healthcare use that appears to be unique to ISs and is not addressed in existing literature is their concerns about continuing the mental health treatments they first received in Scotland once they graduate and return to their home country. The reason why this is unique to ISs is because, unlike in previously researched migrant populations who migrate for an indefinite period of time, ISs' time abroad is temporary, constrained by the duration of their studies. Further investigation is necessary to better understand how ISs' unique migration status influences their healthcare engagement patterns differently from permanent migration experiences.

Transnational help-seeking can also be considered through the lens of liminality. Marotta (2025) re-framing of liminality in context of the migration experience emphasises that early works on liminality by Clay H. Trumbull (1896) describe liminality as a non-linear process where crossing the metaphorical threshold is not final. Instead, migrants physically, psychologically, socially and culturally loop between the pre- and post-liminal (Marotta, 2025). Likewise, in the present study, in order to ensure continuity of care, ISs felt they had to navigate two healthcare systems—one in Scotland and another in their home country—in parallel, crossing the threshold (in this case, the distance between Scotland and their home country) back and forth between the pre- and post-liminal contexts. Considering help-seeking as an adaptive coping strategy (Rickwood & Thomas, 2012), some ISs could be even seen as leveraging the non-linear character of their liminal state to actively resolve their mental distress, re-entering into the liminal state and returning to the pre-liminal context of their home countries. In doing so, they were able to seek mental health support in their home countries when they perceived the services in Scotland as difficult to access or inadequate to effectively address their mental health concerns, pointing to their agency in navigating both their liminal status and help-seeking journeys (Simich et al., 2009). This however was associated with added obstacles, such as liaising between doctors in two countries, translating documents, repeating medical tests, which impeded ISs' help-seeking endeavours. Therefore, the attempts of ISs to seek help for their mental health concerns through the Scottish primary healthcare services were hindered by systemic barriers, some of which are unique to their liminal status as ISs. Given that the present study shows that ISs’ perception of availability of sources of help was not limited to the host country, but also included services available to them in their home country, a case could be made for extending the applicability of Rickwood et al. (2005) conceptualisation of help-seeking to transnational contexts. Additional research is needed to substantiate the possible theoretical implications of transnational help-seeking experiences.

In addition, in line with past research, a major barrier to help-seeking involved ISs' negative mental health attitudes. ISs were aware of their home cultures' negative mental health attitudes (i.e., perceived stigma; Bracke et al., 2019; Tesfaw et al., 2020); with psychological distress seen as “*going mad*” or “*crazy*”. Their accounts of mental health help-seeking unveiled how these perceived stigma beliefs were internalised as personal stigma (Latalova et al., 2014), for instance in how they described psychological distress as “*outlandish*”. These negative culturally-mediated attitudes offer an explanation towards their reluctance in seeking mental health support, whereby their help-seeking was hindered by limited awareness of symptoms, inability to express them and unwillingness to disclose them to the GP (Rickwood et al., 2005). Some ISs reported that were only willing to seek help when their symptoms were “*extreme*” or “*serious*” enough. Even then, they were hesitant to disclose their mental health difficulties, both to friends and families, as well as during the GP appointments. In similar vein, in a qualitative study, Cogan et al. (2024) reported that Asian ISs in Scotland expressed perceived and personal mental health stigma, which was partially why, instead of seeking help for their mental health difficulties, they chose to cope with them on their own. Likewise, quantitative research corroborates that perceived and personal stigma act to hinder ISs' help-seeking endeavours (Bracke et al., 2019; Jennings et al., 2015; Pedersen & Paves, 2014), with culture accounting for higher levels of perceived and personal stigma among ISs, and consequently greater reluctance to seek mental health support in this population (Maeshima & Parent, 2022). Thus, ISs' help-seeking was negatively affected by culturally-mediated attitudinal barriers.

Given that the sample consisted entirely of female ISs, the aforementioned help-seeking barriers may be partly explained by gendered help-seeking patterns. Research shows that women show a greater inclination towards help-seeking compared to other genders, especially in terms of formal help-seeking (Al Azdi et al., 2025; Wendt & Shafer, 2016) and seeking mental health support tends to be seen as more socially acceptable among women (Lee et al., 2024). However, female ISs in the present study did acknowledge and, in some cases, endorse the social “*stigma about speaking*” about mental health which in turn hampered their help-seeking endeavours. Since current literature highlights this pattern of help-seeking as more prevalent among men (Parent et al., 2018; Sheikh et al., 2025), including male ISs (Dombou et al., 2023), research should continue exploring the gender-specific dimensions of ISs' help-seeking experiences.

### Impact of the quality of the patient-GP interaction

ISs' perceptions of the patient-GP interaction revealed an influence of the GP's demeanour and the ISs' help-seeking endeavours. In part, what made them reluctant to seek mental health support through the Scottish primary healthcare services was the perceived quality of the patient-GP interaction itself based on their own experience. Mostly, ISs experienced the patient-GP interaction as devoid of “*human approach*”, with the GPs responding to their mental health concerns in a “*cold*”, “*impersonal*”, “*rushed*” and “*dismissive*” manner. In turn, this led to ISs feeling “*discouraged*” from seeking mental health support from Scottish primary healthcare services in the future. Similarly, Tunks et al. (2023) found that patients' perceptions of primary healthcare settings and medical interactions with the GP affected their help-seeking journeys. Just as in the present study, the experiences of patients with common mental health problems were punctuated by feeling rushed, not understood or shown empathy, dismissed. As such, they described primary healthcare services as “*not the place for common mental health disorders*” (Tunks et al., 2023, p.334), leaving patients disappointed with the level of care and offered treatments, and so creating a barrier for them so seek further mental health support. Meanwhile, Parker et al. (2020a) found that a patient-GP interaction characterised by warmth, empathy and a sense of human connection was one of the main facilitators in patients' help-seeking endeavours. Therefore, the present study, alongside previous literature, points towards the importance of GPs' demeanour in shaping ISs' perceptions of the patient-GP interaction, and consequently their help-seeking endeavours.

On one hand, the perception of GP's demeanour as lacking warmth and empathy can be explained by the time-limited nature of the patient-GP interaction. In the UK, GP appointments are designed to be 10−15 minutes long (British Medical Association, 2025), and this short duration might enforce symptom-oriented conversations which can come across as “*cold*”, “*impersonal*” and “*rushed*”. ISs experiential accounts attributed the GP’s demeanour to professional stigma, i.e., the mental health stigma exhibited by healthcare professionals towards their patients (Subu et al., 2021). This aligns with Link and Phelan (2001) conceptualisation of stigma in terms of five interrelated components: labelling, stereotyping, separation, status loss and discrimination, and power, all of which were present in this study. ISs' description of negative mental health attitudes that they perceived to be prevailing within the Scottish primary healthcare services, especially their feeling that mental health difficulties were not seen as serious relative to physical health concerns, are indicative of labelling and stereotyping, whereby having a mental health disorder became a salient social marker influencing GPs' clinical judgement and attitudes. This sense of divide between patients with physical health concerns and those experiencing mental health difficulties (i.e., “*separation*” in Link & Phelan, 2001) led to status loss and discrimination. The negative attitudes expressed by GPs within medical interactions meant that most ISs experienced these interactions as unempathetic and less thorough, with most ISs in this study feeling that, in contrast to physical health concerns, their mental health struggles were dismissed as not serious enough to justify preferred treatment. According to these ISs, these expressions of professional stigma impacted ISs' access to resources, treatment options, and overall experience (i.e., “*power*” in Link & Phelan, 2001), explaining their subsequent reluctance to seek help via the Scottish primary healthcare services. Consequently, perceptions of professional stigma emerged as a notable barrier to help-seeking for ISs within the Scottish primary healthcare system (Henderson et al., 2013; Pingitore et al., 2001).

It is possible that the GPs' behaviours and attitudes perceived by ISs as professional stigma may be attributed to institutional frameworks. For instance, Parker et al. (2020b) reported that GPs from the English primary healthcare services were highly emphatic of the role that building rapport, being supportive and providing person-centred care for patients experiencing psychological distress. However, they felt that time pressure and institutional constraints prevented them from implementing this in patient consultations. The GPs were particularly critical of the Quality of Outcomes Framework (NHS England, 2024) which, by prioritising the use of screening questions and a stepped-care approach, has been previously described as a hindrance to patient-led consultations and listening to patient concerns (Maisey et al., 2008; Parker et al., 2020b). Likewise, Dutch GPs described how protocols and guidelines pose a barrier to empathetic behaviour towards patients, hindering genuine reactions and interest (Derksen et al., 2016). Indeed, over the years, protocol-driven guidelines may have led to a shift from listening and empathy to task-oriented communication, with primary care consultations being increasingly somatically oriented (Butalid et al., 2014).

Overall, whereas past research has given much attention to the impact of the IS-specific factors on help-seeking, this study highlights how help-seeking is in fact a dyadic process, whereby healthcare professionals play an active role in shaping ISs' help-seeking trajectories. Further research should explore the potential effect of professional stigma on help-seeking, particularly its impact on the perceived quality of patient-GP interaction. To account for the aforementioned dyadic character of help-seeking, prospective studies should include both patient and GP voices. Such research should also take into account the ways in which institutional frameworks can unintentionally reinforce professional stigma within medical encounters (Parker et al., 2020b; Subu et al., 2021). A mixed-methods approach would be particularly beneficial, allowing to systematically quantify the relational dynamics identified in the present study. This, in conjunction with healthcare sociology and organisational psychology literature, would allow to extend help-seeking frameworks to consider the potential role that relational dynamics experienced within medical encounters can play in facilitating or hindering continued engagement with help-seeking and its outcomes.

### Limitations

The sample consisting entirely of female ISs is a significant limitation. Acculturation and acculturative stress contribute to psychological distress differently for male, female and non-binary ISs (Castillo et al., 2015). Likewise, as discussed above, the underutilisation of mental health services by ISs is also a gendered phenomenon, with male ISs less likely to seek mental health support compared to females (Eisenberg et al., 2007; Xiong & Yang, 2021). While this research speaks to the experiences of its participants and is not pursuing generalisability beyond the described context, future work could explore the gendered nature of acculturation, acculturative stress and help-seeking among ISs.

Although the present study included participants from a wide range of cultural backgrounds, these were homogenised under the umbrella term of ISs. As such, the study offers only a limited insight into culturally mediated barriers to help-seeking, including mental health stigma. With the level and nature of mental health stigma varying between cultures (Gopalkrishnan, 2018; Koschorke et al., 2017), it is possible that the impact of mental health stigma on ISs' help-seeking differs depending on their home cultures. That said, the present study focused on exploring help-seeking experiences, not through the lens of participants' diverse cultural backgrounds, but rather through their shared identity as ISs.

### Implications

The present study offers novel theoretical insights regarding female young adult migrant mental health by advancing the concept of dual liminality to articulate the unique, intersecting and, at times, challenging experiences of migration and emerging adulthood, as well as their transformative potential. Such perspective captures how young adult migrants, such as ISs, simultaneously navigate multiple ‘in-between’ states—culturally, caught between their origin and host societies, and developmentally, transitioning into adulthood. This nuanced framing reveals how social, cultural, financial, and psychological factors intertwine to create distinctive challenges which may lead to psychological distress, as well as affect if and how help is sought for these difficulties. This theoretical lens directs research and practice to assess, target, and support these liminal processes through interventions that are both culturally responsive and developmentally attuned. Though the present study, due to its idiographic commitment, was not striving for generalisability, further research should consider this phenomenon among a more gender-diverse sample.

In practical terms, this study emphasises the need for universities to employ strategies to mitigate the acculturative stress experienced by ISs (Castillo et al., 2015). Through strategies such as information sessions, dedicated advisory teams or peer support, universities should facilitate ISs transition to living and studying abroad by offering support in the academic, social and financial domains, whilst staying attuned to the cultural differences and sense of isolation experienced specifically by ISs upon migrating to a foreign country. As mentioned, these approaches should also take into account the significant developmental transitions that young adult ISs face. Should ISs experience psychological distress due to their circumstances, universities should encourage help-seeking through equipping them with the support and knowledge required to navigate the NHS. These steps would enable universities to even further advance their adoption of equality, diversity and inclusion values beyond learning and teaching, holistically enhancing educational experience for students from all backgrounds (Gunn et al., 2015). Moreover, considering the reports of economic “*reliance*” of UK universities on ISs and the economic contribution they make (House of Lords Industry and Regulators Committee, 2023; Lapworth, 2024), universities should take measures to prioritise IS mental health and wellbeing of this population in order to prevent dropout (Bradley, 2017; Brennan, 2022; Neves et al., 2024).

Given the role of patient-GP interactions in ISs' help-seeking trajectories, primary healthcare institutions should prioritise initiatives to improve the quality of these interactions. Evidence suggests that mental health training among GPs is an effective strategy to address potential professional stigma, thus improving the quality of patient-GP interactions and treatment outcomes (Caulfield et al., 2019). Considering the culturally situated nature of ISs' psychological distress and help-seeking endeavours, training should foster cultural competence among GPs, i.e., understanding how culture might influence mental health needs and outcomes (Lok, 2022). Although already embedded within medical education curricula (General Medical Council, 2003), such training should recognise one's learning through informal and hidden curricula, i.e., cultural influences received in the general institutional environment (Liu et al., 2022). This in turn emphasises the need for not just medical education settings but healthcare institutions more broadly to strengthen their day-to-day support for equality, diversity and inclusion, starting with improved infrastructure and resources to support the delivery of cultural competency training as a part of continuous professional development. Furthermore, institutional frameworks need to be evaluated, and where necessary modified, to enable GPs to provide more patient-centred care, reducing barriers created by time constraints and rigid protocols. For example, based on the present study, policy-level adaptations to allocate resources for longer, more tailored consultations could contribute to fostering sustainable patient-centred care. By doing so, healthcare services can create a more supportive environment that encourages ISs to seek necessary mental health support.

## Conclusions

This IPA study aimed to qualitatively explore ISs' experiences of help-seeking experiences via the Scottish primary healthcare services, whilst living and studying in Scotland. Findings from in-depth interviews with nine female ISs who had recently sought mental health support from the Scottish primary healthcare services, though limited to a gender-homogenous sample, showcase the unique challenges that ISs face when living and studying in a foreign country and their mental health impact.

ISs' help-seeking journeys were marked by barriers—culturally-mediated attitudes towards mental health, difficulties accessing and navigating healthcare in a transnational context, and experiences of professional stigma expressed by GPs within patient-GP interactions. These findings underscore how, although affected by individual factors, help-seeking is not a one-sided process. Instead, help-seeking trajectories may be equally affected by the perceived quality of patient-doctor interactions along with the accessibility of the broader healthcare system. Insights from this study can be used by universities to develop targeted interventions aimed at improving the mental wellbeing of ISs, with the long-term goal of increasing IS enrolment and retention rates due to enhanced student experience. The findings can also be considered by primary healthcare institutions to improve the quality of patient-GP interactions.

## Supplementary Material

Supplementary material Supplementary Material 1

## Data Availability

Data associated with the paper is not publicly available as the participants of the study did not give written consent for their raw data to be shared publicly. Any further data will be made available upon reasonable request.
