# Supplementary material for: “You feel hopeless when you can't access healthcare”: International students' experiences of mental health help-seeking through primary healthcare services in Scotland
Source: Int J Qual Stud Health Well-being. 2025 Nov 2;20(1):2572518. doi: 10.1080/17482631.2025.2572518 (PMC12581769; doi:10.1080/17482631.2025.2572518)
Supplement: Supplementary material — 1 [file ZQHW_A_2572518_SM1808.docx]

**Supplementary material 1: Interview schedule**

**Pre-interview**

Before we start, I just want to remind you that you can share with me as much or as little as you wish. I will not ask explicitly about your mental health history, and you do not need to disclose that. Instead, I will ask you to describe how you sought help and what that was like for you. We can take as many breaks as often as you need.

I want to learn more about your experiences of seeking help for your mental health at the local primary care services, more specifically your thoughts, feelings and perceptions before, during and after the appointment. I am also interested in your perspective on the doctor’s approach and demeanour during the appointment and the quality of help you were offered.

**Opening questions**

Tell me a bit about yourself. How long have you lived in [city]?

What are your experiences of living here and attending a university?

How are you finding studying abroad so far?

**Before the appointment**

Please could you tell me why you decided to reach out to your GP practice for help?
*Prompts: Why now? Why haven’t you done so before? Were you hesitant?*

How did you know that GP practice is a place to look for help for you mental health concerns?

*Prompts: Have you looked elsewhere before? Was the guidance sufficient? If not, what would you change about it?*

Could you talk me through your experience of setting up your first GP appointment? What was that like for you?

What were your expectations when reaching out to the GP for the first time?

*Prompts: What were you expecting from the first appointment? Did you have any concerns?*

**During the appointment**

Could you talk me through what happened during the first appointment?

*Prompts: Could you elaborate on that?*

How did you feel during this appointment? *Prompts: Why? What do you mean by that?*

What was your experience of sharing your mental health concerns with the doctor for the first time? *Prompts: What did it feel like? How did the doctor react?*

How would you describe the doctor’s behaviour during the appointment? How did they make you feel?
*Prompts: Could you give me some examples of doctor’s behaviours that made you feel good? Could you give me some examples of doctor’s behaviours that made you feel bad?*

What help/solutions for your mental health difficulties were offered to you by the GP during the first appointment? What do you think about that?
*Prompts: What was the help/solution you were looking for? What help/solution were you expecting? Why?*

**After the appointment**

How did you feel after that first appointment?

Looking back at your experience, was the first appointment beneficial?
*Prompts: If not, why? What would you change anything about it? If yes, what was beneficial about it?
What solutions were you offered? In what way did the appointment help you?*

Have you gone back to your GP since the first appointment? Are you planning on going back in the future?
*Prompts: If not, why? If yes, could you tell me what did the subsequent appointments look like? / What are your expectations about the future appointments?*

During your subsequent appointments, how did the GP react to your mental health concerns? How did they act?

Overall, looking back at your experiences before, during and after appointments, what are your perspectives and opinions of the UK primary mental healthcare so far?

*Prompts: Do you see any areas that could be improved upon? If so, what are they? How could they be improved?*

Ideally, (during your future appointments) how do you think doctors should behave/what should they do when their patients tell them about their mental health struggles?
*Prompts: Why? Could you explain that further? Why do you think that’s important? How does that compare to how the doctors actually behaved during your appointments?*

**Post-interview**

Thank you for taking the time to meet with me today. Before we finish, I just wanted to ask how are you feeling after the interview? If you want to discuss how you're feeling with someone other than myself, you can find contact information for free counselling & psychological services within the [university] as well as external mental health helplines on this sheet (hand them the Debriefing Sheet).

I will soon be in touch via the email address you gave me to arrange the reimbursement. In case of any questions, relevant contacts are listed on the Participant Information Sheet which I gave you at the beginning of the session. If you are interested, in September, I will email you a report summarising the findings of the study.
